# Heterogeneity in Methods of Estimating Kidney Function for Cancer Clinical Trial Eligibility

**DOI:** 10.1001/jamanetworkopen.2024.33387

**Published:** 2024-09-16

**Authors:** Alexander B. Karol, Rodrigo Paredes, Yu Fujiwara, Anna Argulian, Himanshu Joshi, Matthew Abramson, Matthew D. Galsky

**Affiliations:** 1 Department of Medicine, Icahn School of Medicine at Mount Sinai, Mount Sinai Hospital, New York, New York; 2 Department of Medicine, Icahn School of Medicine at Mount Sinai, Mount Sinai Beth Israel, New York, New York; 3 Department of Medicine, Roswell Park Comprehensive Cancer Center, Buffalo, New York; 4 Icahn School of Medicine at Mount Sinai, New York, New York; 5 Division of Hematology and Medical Oncology, The Tisch Cancer Institute, Icahn School of Medicine at Mount Sinai, New York, New York; 6 Department of Population Health and Policy, Icahn School of Medicine at Mount Sinai, New York, New York; 7 Division of Nephrology, Icahn School of Medicine at Mount Sinai, New York, New York

## Abstract

This cross-sectional study examined the kidney-function eligibility criteria for patients for enrollment in contemporary cancer clinical trials.

## Introduction

Kidney function is frequently impaired in patients with cancer due to preexisting chronic kidney disease, cancer-associated factors, and prior anticancer treatments. Given these multifactorial insults, accurate assessment of kidney function is critical to determining patient eligibility for clinical trial enrollment. Measurement of the glomerular filtration rate (GFR) using clearance of exogenous markers provides the most accurate measure of kidney function but is not practical clinically. Although serum creatinine (sCr) level alone has been used to estimate kidney function, it can be influenced by non-GFR physiologic determinants, such as sarcopenia, and nephrology and oncology organization guidelines recommend against using sCr level alone.^1^ Several equations incorporating clinical and demographic variables have been developed to improve the estimation of kidney function.^2^ Recently, there have been calls for harmonization of most aspects of cancer clinical trial eligibility to foster feasibility, inclusivity, comparability, and generalizability.^3^ We sought to understand the landscape of approaches used to estimate kidney function for contemporary cancer clinical trial eligibility.

## Methods

In this cross-sectional study, ethics approval and consent to participate were not required because the institutional review board of the Icahn School of Medicine did not qualify this study as human research. We systematically searched the ClinicalTrials.gov database to review trial demographics and kidney eligibility criteria for phase 3 clinical trials. Trials were included if completed between October 28, 2013, and October 28, 2023, and if anticancer drugs for adults were evaluated. Trial demographics and methods of kidney function estimates used for trial eligibility were recorded (eMethods in Supplement 1). Descriptive statistical analysis was performed using R, version 4.3.2. This study followed the STROBE reporting guideline.

## Results

A total of 436 trials met criteria for review, and 231 trials, enrolling 111 424 patients, met the criteria for inclusion. Characteristics of the included trials are shown in the Table; 139 of 197 (70.6%), 35 of 231 (15.1%), and 200 of 231 (86.5%) trials used the Cockcroft-Gault (CG) formula, sCr level alone, or either CG or sCr level to define kidney function eligibility, respectively (Figure, A and B). Trends in methods used to estimate kidney function for trial eligibility over time are shown in the Figure, C.

**Table.** Characteristics of Phase 3 Clinical Trials

Trial characteristic	No. (%)	
	Trials (n = 231)	Patients (n = 111 424)
Cancer type		
Breast	28 (12.1)	18 295 (16.4)
Gastrointestinal	43 (18.6)	21 216 (19.0)
Genitourinary	22 (9.5)	13 816 (12.4)
Gynecologic	12 (5.2)	5831 (5.2)
Head and neck	14 (6.1)	7055 (6.3)
Hematologic	46 (19.9)	12 558 (11.3)
Lung	54 (23.4)	27 406 (24.6)
Skin	12 (5.2)	5247 (4.7)
Treatment		
All targeted trials	106 (45.9)	51 824 (46.5)
All IO trials	103 (44.6)	54 318 (48.7)
All chemotherapy trials	91 (39.4)	46 175 (41.4)
Targeted monotherapy	69 (29.9)	29 472 (26.5)
IO monotherapy	55 (23.8)	28 779 (25.8)
IO + chemotherapy	38 (16.5)	21 548 (19.3)
Targeted therapy + chemotherapy	37 (16.0)	18 361 (16.5)
Chemotherapy monotherapy	16 (6.9)	6266 (5.6)
IO + targeted therapy	10 (4.3)	3991 (3.6)
Endocrine	2 (0.9)	646 (0.6)
Biologic	2 (0.9)	601 (0.5)
Other	2 (0.9)	1760 (1.6)
Therapy		
Monotherapy	107 (46.3)	42 471 (38.1)
Combination	124 (53.7)	68 953 (61.9)
Funding source		
Industry	227 (98.3)	110 934 (99.6)
Government	4 (1.7)	490 (0.4)
Trial enrollment, No.		
<100	19 (8.2)	1039 (0.9)
100-200	36 (15.6)	5058 (4.5)
201-500	80 (34.6)	28 296 (25.4)
501-1000	80 (34.6)	54 221 (48.7)
>1000	16 (6.9)	22 810 (20.5)
Enrollment start year		
2013-2015	103 (44.6)	53 825 (48.3)
2016-2018	119 (51.5)	53 960 (48.4)
2019-2022	9 (3.9)	3639 (3.3)
Race^a^		
Black	151 (65.4)	1818 (1.6)
Not available	28 (12.1)	9607 (8.6)
Other^b^	185 (80.1)	29 347 (26.3)
White	176 (76.2)	70 652 (63.4)

Abbreviation: IO, immunotherapy.

^a^ Trials with at least 1 patient.

^b^ Defined as any reported race or ethnicity for a specific study that was not Black or White.

**Figure.**
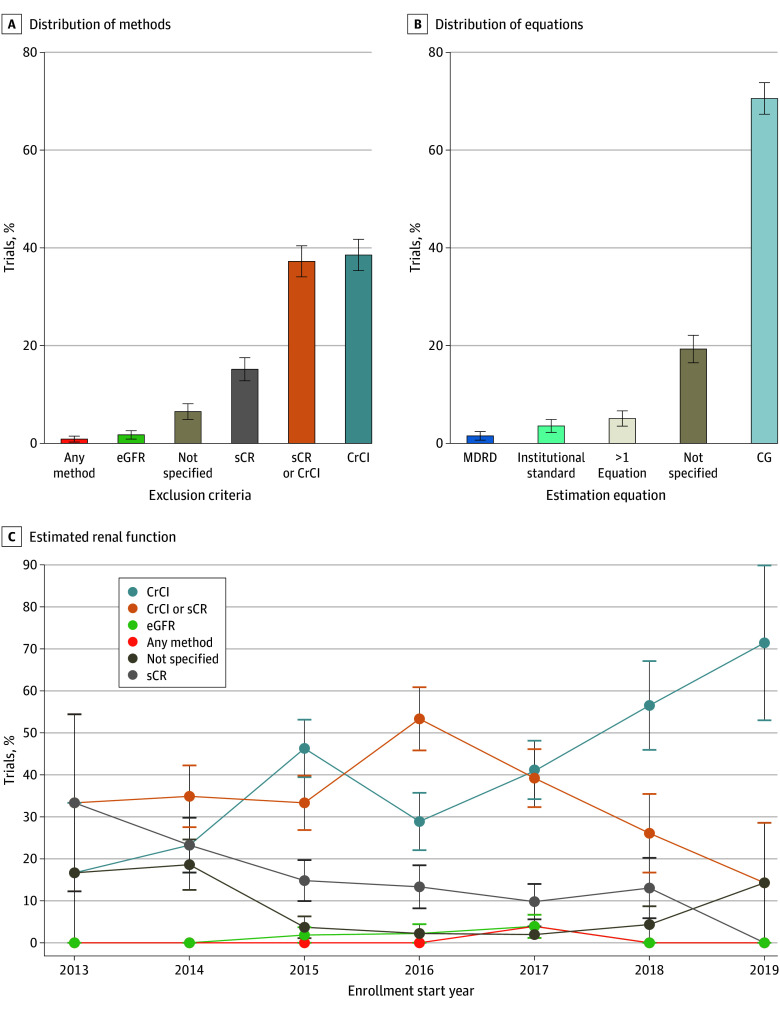
Approaches to Estimate Kidney Function to Define Eligibility in Contemporary Phase 3 Cancer Clinical Trials Distribution of methods (A) and equations (B) to estimate kidney function. C, Overall distribution of the kidney function estimation approach according to the enrollment start year. Trials using serum creatinine (sCr) or creatinine clearance (CrCl) level specified that patients could be eligible by meeting either criteria. Vertical bars indicate 1 SE. CG indicates Cockcroft-Gault; eGFR, estimated glomerular filtration rate; and MDRD, Modification of Diet in Renal Disease.

## Discussion

Among 231 phase 3 cancer clinical trials in our analysis, we observed substantial heterogeneity in the methods used to estimate kidney function for clinical trial eligibility. Although professional societies recommend against its use, and despite an improved trend in recent years, 11 of 32 (34.4%) of cancer clinical trials since 2018 still use sCr level alone to define kidney function eligibility.^1,3,4^ The CG formula was the most frequently used equation to estimate kidney function. The CG formula was developed in 1973 using data from 249 men and is known to underestimate kidney function in patients with cancer, and nephrology societies recommend modern approaches to estimate the GFR.^1^ Contemporary measures, such as the Chronic Kidney Disease–Epidemiology Collaboration (CKD-EPI) and Modification of Diet in Renal Disease equations, were used in a few trials. A limitation of our study, however, is that we included only phase 3 trials, which often require less precise kidney function measurements than earlier phase trials.

Despite calls for harmonized clinical trial eligibility across multiple domains, no uniform guidelines currently exist regarding estimation of kidney function. Although various anticancer agents necessitate distinct kidney function thresholds based on their metabolism and toxicity profiles, standardization of methods to estimate kidney function for trial eligibility would foster a comprehensive understanding of the effect of kidney function on adverse events and cancer-related outcomes across clinical trials. Despite momentum to preferentially use the race-free CKD-EPI 2021 formula to estimate GFR, a preferred calculation to estimate kidney function is not well defined.^1^ Certain anticancer drugs can also impair tubular secretion of sCr, making accurate estimation of the GFR more challenging.^5^ Although cystatin C is another readily available biomarker that may improve GFR estimation, there is concern that certain cancers may affect cystatin C levels endogenouslly.^6^ Together, our findings call for a collaborative effort among key stakeholders to establish a standardized approach to estimate kidney function to define cancer clinical trial eligibility.

## Notes

Supplement 1.
eMethods.

Supplement 2.
Data Sharing Statement
