# Supplementary material for: Heterogeneity in Methods of Estimating Kidney Function for Cancer Clinical Trial Eligibility
Source: JAMA Netw Open. 2024 Sep 16;7(9):e2433387. doi: 10.1001/jamanetworkopen.2024.33387 (PMC11406393; doi:10.1001/jamanetworkopen.2024.33387)
Supplement: Supplement 1. — eMethods. [file jamanetwopen-e2433387-s001.pdf]

Supplemental Online Content

Karol AB, Paredes R, Fujiwara Y, et al. Heterogeneity in methods of estimating kidney function for cancer clinical trial eligibility. *JAMA Netw Open*. 2024;7(9):e2433387. doi:10.1001/jamanetworkopen.2024.33387

eMethods.

This supplemental material has been provided by the authors to give readers additional information about their work.

© 2024 Karol AB et al. *JAMA Network Open.*

eMethods.

Three reviewers (AK, RP, AA) independently reviewed the clinical trials. Patient race and ethnicity data was obtained using the clinicaltrials.gov database. Race and ethnicity options included "American Indian or Alaska Native", "Asian", "Black or African American", "More than one race", "Native Hawaiian or Other Pacific Islanders", "White", "Unknown or Not Reported." "Other" was defined as any reported race or ethnicity for a specific study that was not "Black or African American", nor "White," Race and ethnicity were assessed to provide a comprehensive overview of the patient populations included in cancer clinical trials and to highlight the proportion of the population eligible for racial correction factors included in some equations to estimate renal function (e.g. CKD-EPI 2009).

© 2024 Karol AB et al. *JAMA Network Open.*
