# Supplementary material for: Heterogeneity in Methods of Estimating Kidney Function for Cancer Clinical Trial Eligibility
Source: JAMA Netw Open. 2024 Sep 16;7(9):e2433387. doi: 10.1001/jamanetworkopen.2024.33387 (PMC11406393; doi:10.1001/jamanetworkopen.2024.33387)
Supplement: Supplement 2. — Data Sharing Statement [file jamanetwopen-e2433387-s002.pdf]

Data Sharing Statement

Karol. Heterogeneity in Methods of Estimating Kidney Function for Cancer Clinical Trial Eligibility. *JAMA Netw Open*. Published September 16, 2024.
doi:10.1001/jamanetworkopen.2024.33387

Data
Data available: Yes
Data types: Data (not involving human participants)
How to access data: 10.6084/m9.figshare.25565643 alexanderb95@gmail.com
When available: With publication

Supporting Documents
Document types: Statistical/analytic code
How to access documents: 10.6084/m9.figshare.25565643 alexanderb95@gmail.com
When available: With publication

Additional Information
Who can access the data: Researchers whose proposed use of the data has been approved
Types of analyses: Any purpose
Mechanisms of data availability: with investigator support
